# Measurement Time Reduction by Means of Mathematical Modeling of Enzyme Mediated RedOx Reaction in Food Samples Biosensors

**DOI:** 10.3390/s21092990

**Published:** 2021-04-24

**Authors:** Arantzazu Florez, Elena Murga, Itziar Ortiz de Zarate, Arrate Jaureguibeitia, Arkaitz Artetxe, Basilio Sierra

**Affiliations:** 1 Vicomtech Foundation, Basque Research and Technology Alliance (BRTA), Mikeletegi 57, 20009 Donostia-San Sebastián, Spain; aartetxe@vicomtech.org; 2 Department of Computer Sciences and Artificial Intelligence, University of the Basque Country (UPV/EHU), 20018 Donostia-San Sebastián, Spain; b.sierra@ehu.eus; 3 Biolan Microbiosensors S.L., Parque Tecnológico de Bizkaia, Laida Bidea 409, 48170 Zamudio, Spain; emurga@biolanmb.com (E.M.); iortizdezarate@biolanmb.com (I.O.d.Z.); ajaureguibeitia@biolanmb.com (A.J.)

**Keywords:** chemical and biological sensors, mathematical modeling, optimization, kinetic modeling, parameter estimation

## Abstract

The possibility of measuring in real time the different types of analytes present in food is becoming a requirement in food industry. In this context, biosensors are presented as an alternative to traditional analytical methodologies due to their specificity, high sensitivity and ability to work in real time. It has been observed that the behavior of the analysis curves of the biosensors follow a trend that is reproducible among all the measurements and that is specific to the reaction that occurs in the electrochemical cell and the analyte being analyzed. Kinetic reaction modeling is a widely used method to model processes that occur within the sensors, and this leads to the idea that a mathematical approximation can mimic the electrochemical reaction that takes place while the analysis of the sample is ongoing. For this purpose, a novel mathematical model is proposed to approximate the enzymatic reaction within the biosensor in real time, so the output of the measurement can be estimated in advance. The proposed model is based on adjusting an exponential decay model to the response of the biosensors using a nonlinear least-square method to minimize the error. The obtained results show that our proposed approach is capable of reducing about 40% the required measurement time in the sample analysis phase, while keeping the error rate low enough to meet the accuracy standards of the food industry.

## 1. Introduction

An amperometric biosensor is based on a biological detection element coupled to a physical-chemical transducer that converts the biological signal, originated by the interaction between this detection element and the analyte, into a quantitative result. The biological element recognizes the target to be analyzed selectively and this interaction is translated into current by the transducer [1]. Amperometric detection consists of a polarization of the electrode by a fixed potential and recording the current due to the electrochemical transformation (RedOx reaction) of the targeted analyte [2].

There are three types of amperometric biosensors [3]:

1. First-generation biosensors: The obtained signal is due to the electrochemical reaction of the reactive agent, which is involved in the biochemical transformation of the target compound.
2. Second-generation biosensors: It is characterized by the use of a mediator. The obtained current is the result from the oxidation/reduction of the mediator.
3. Third-generation biosensors: The result is based on the direct electron transfer mechanism to the transducer.

**Figure 1:**
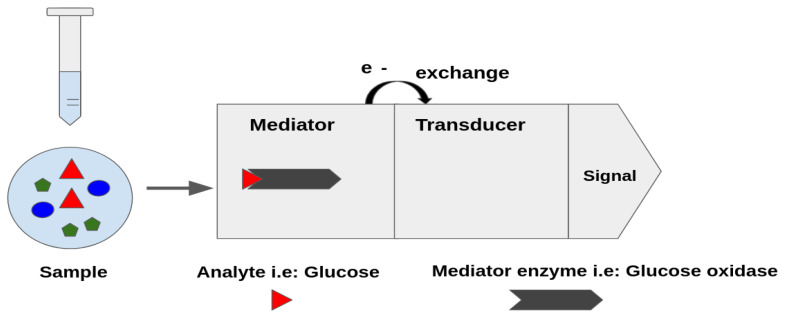
Scheme of an example of an amperometric glucose biosensor.

**Figure 2:**
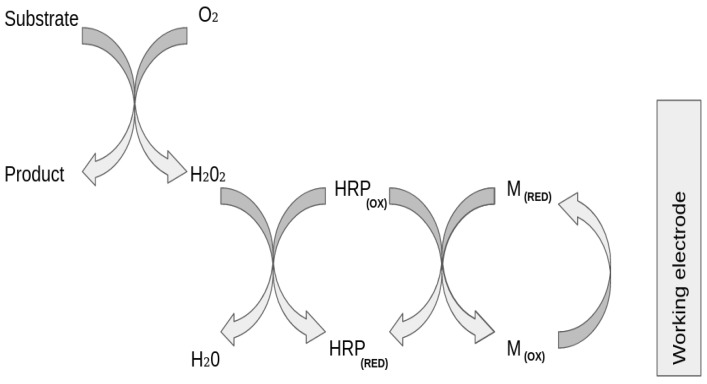
Schematic representation of the RedOx reaction.

In the case of second-generation biosensors, which are the ones this paper focuses on, their main characteristic is that they are based on the use of RedOx mediators. In Figure 1, an example of an amperometric biosensor is presented where the mediator is the glucose oxidase that acts as the catalysts of the RedOx reactions allowing the electron transfer [4]. This release of electrons is proportional to the amount of glucose present in the sample, which would be detected by the transducer and turned into current. Once the sample is added in the medium, the Redox reaction begins, the enzyme that is immobilized in the biosensor interacts specifically with the analyte, catalyzing its oxidation or reduction, as depicted in Figure 2. The enzyme that is specific to the analyte mediates the oxidation of the substrate to the product, generating hydrogen peroxide in the presence of oxygen. The current obtained is the representation of the indirect measurement of the analyte that is caused by the catalytic reduction of the hydrogen peroxide at the working electrode.

**Figure 3:**
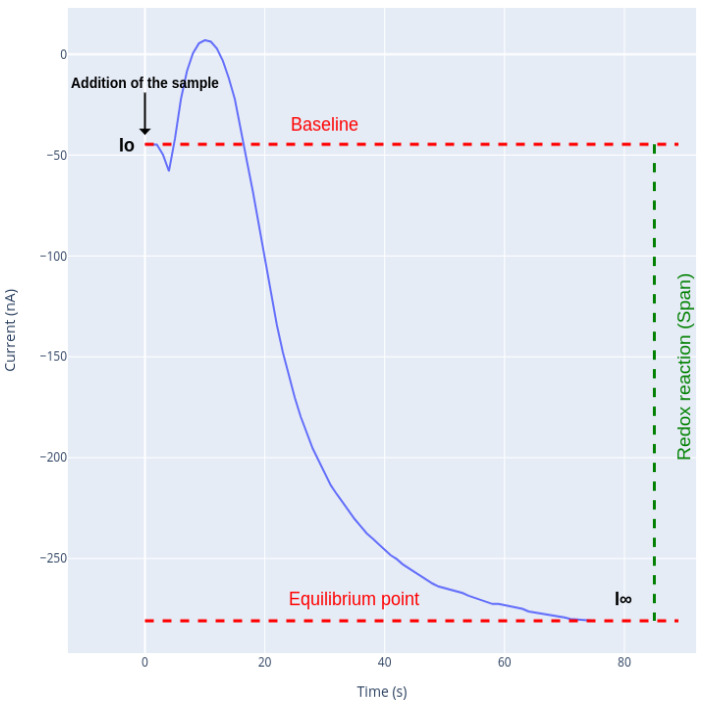
Temporal response of the biosensor.

The analyte concentration is directly proportional to the RedOx reaction that results in a change in the current, as can be seen in Figure 3. The change in the intensity that can be observed between $I_{o}$ (initial value of the current) and $I_{\infty }$ is called span and depends on the amount of analyte present in the sample; thus, the span value is higher if the sample is more concentrated. $I_{o}$ refers to the electric current produced by the system configuration (applied potential, counter and working electrode, buffer specifications, etc.) before applying any sample. Once the sample is added to the measurement cuvette (or electrochemical cell), the analyte reaches the surface of the working electrode and therefore is oxidized by the enzymes that are immobilized on top of it. This causes a release of electrons, proportional to the amount of sample oxidized, thus altering the electric current of the system, as shown in Figure 3, and the current starts to decay. In our case, as the analysis is performed under stirring conditions, the predominant analyte transport process is convection, from the bulk solution to the surface of the working electrode. This makes the concentration of the non oxidized analyte at the surface of the electrode to remain constant, producing a stable electric current. As the reaction progresses, in the surroundings of the electrode, an equilibrium is achieved between the analyte reaching this area by agitation and the analyte consumed by the enzymatic reaction; this point of the current corresponds to $I_{\infty }$.

Biosensors are devices with great potential in food industry since they can provide information that conventional techniques do not offer.

Analytical methods such as chromatographic techniques, spectrophotometric or electroanalytical methods [5] are some other methods found in the literature for food safety parameter quantification. However, those techniques are usually tedious and expensive, while biosensors are presented as a user-friendly alternative that can improve analysis time by eliminating laborious and costly procedures. Due to their high specificity, selectivity and accuracy, biosensors have great potential in food industry. Furthermore, one of their remarkable features is that they can be used in situ, which allows them to be integrated in all stages of the quality control of food production [6].

A wide variety of enzyme biosensors can be found in the literature based on electrochemical detection that analyze different analytes in food (e.g., glucose, malic acid and lactate) [1,7,8]. Biosensors are designed to perform many measurements, being reproducible among all measurements one of the most important requirements. The reactions that occur within the electrochemical cell are enzyme-based reactions that follow the same curve shape.

Previous studies have shown that kinetic reaction modeling is a widely used method in food industry [9,10,11,12]. For this reason, the possibility of mathematically modeling the response obtained with the biosensor has been raised, which leads to the possibility of improving the efficiency of the analysis and shortening times of measurements. The development of such an algorithm would reduce not only the measuring time but also the costs. The process would be more dynamic and the waiting time between analyses would be reduced, which can lead to a reduction of errors. On the other hand, being more dynamic enables the operator to do more measurements in less time, which could also be a source of cost saving.

The main objective of the proposal is to design a methodology for the improvement of the analysis protocols, achieving biosensor based food safety testing that performs the measurements in less time.

**Figure 4:**
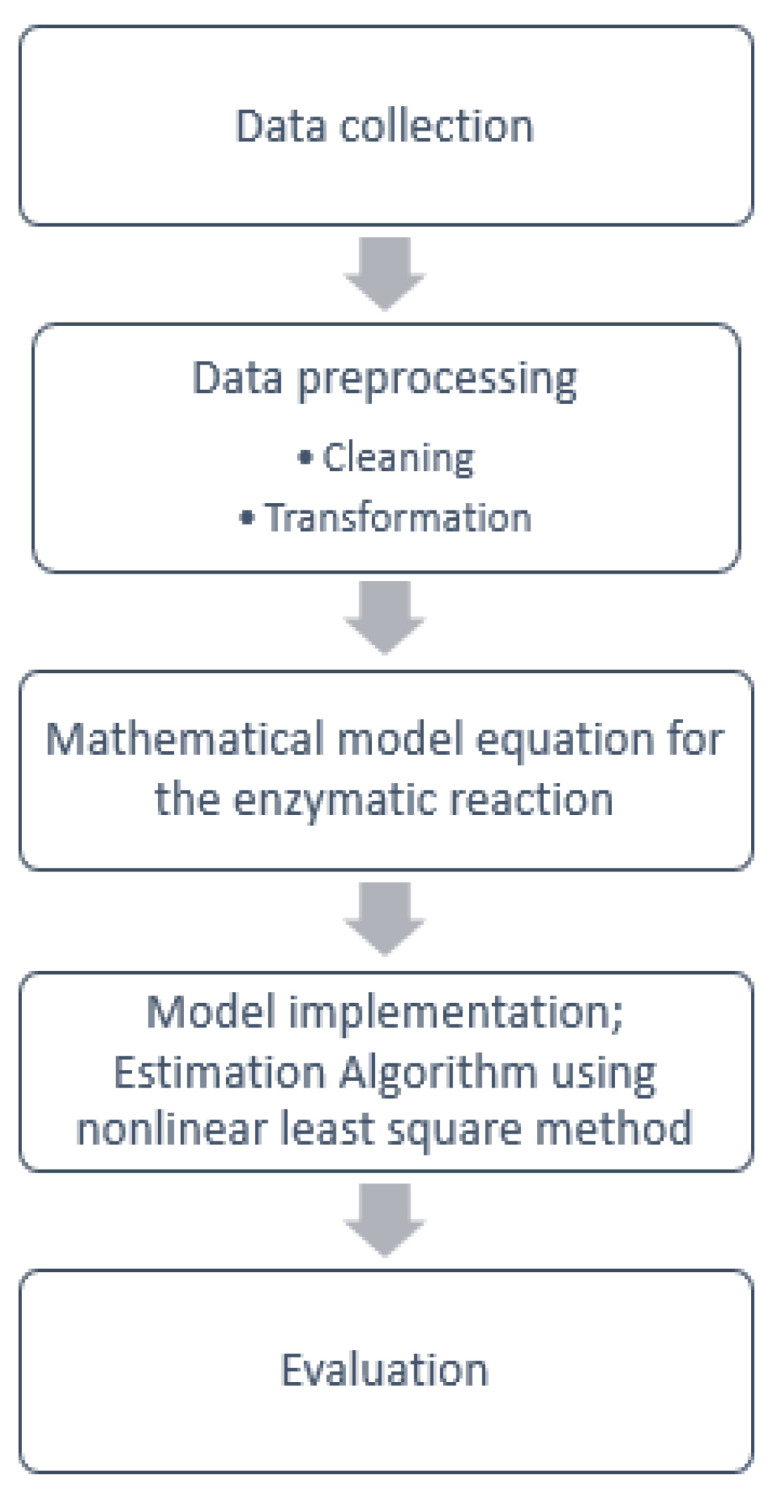
Block diagram for the proposed approach.

To implement a model that allows a reduction in the analysis, the following steps, as summarized in the block diagram presented in Figure 4, were carried out.

By observing the behavior of the analysis (Figure 3), it was seen that the enzymatic reduction reaction resembles an exponential reaction. As the reaction proceeds, the activity of the enzyme decreases due to the fact that the substrate is consumed.

Exponential decrease equations model many chemical and biological processes [9,13,14,15,16], since in many cases the processes depend on the speed and the amount of sample that is present. These types of models are used whenever the velocity at which the reaction happens is proportional to the amount of existing sample. The parameters of the exponential models describe how fast the process occurs, and, hence, the proposed model has a negative exponential function in addition to the adjustment error term. The obtained mathematical model that is a nonlinear regression model that fits the curve of the amperogram output, in order to anticipate the final current measurement. The function to fit is not linear in its parameters, so an iterative algorithm is proposed, specifically, the iterative procedure of Levenberg–Marquardt (LM) [17,18], an algorithm that combines two numerical minimization algorithms: the gradient descent method and the Gauss–Newton method.

The proposed approach obtains a mean reduction of 25 s per test, which means a total saving of 40% with respect to the process now being used, hence achieving a significant reduction in time that would optimize food safety testing in food industry.

The rest of the paper is structured as follows. Section 2 describes the different data processing methods that were used to prepare the data and also summarizes the method used to develop the model that reduces the analysis time. In Section 3, the results of our model are presented, including the filtering parameter selected and the length of the buffer necessary for a good approximation. Section 4 summarizes the conclusions.

## 2. Material and Methods

This section presents the different methodologies that have been used to process the data. The data were obtained by a biosensor, whose technology is based on the application of constant potential on the electrochemical cell. The analyzed data consist of continuously measured current data resulting from the oxidation or reduction of the analyte. The data present noise and apparently random behavior at the beginning of the analysis, thus the data were treated before proceeding to the implementation of the algorithm. For the implementation of the model, LM algorithm was chosen, a nonlinear regression model that consists of estimating the parameters that describe the behavior of the enzymatic reaction.

### 2.1. Amperometric Measurements

**Figure 5:**
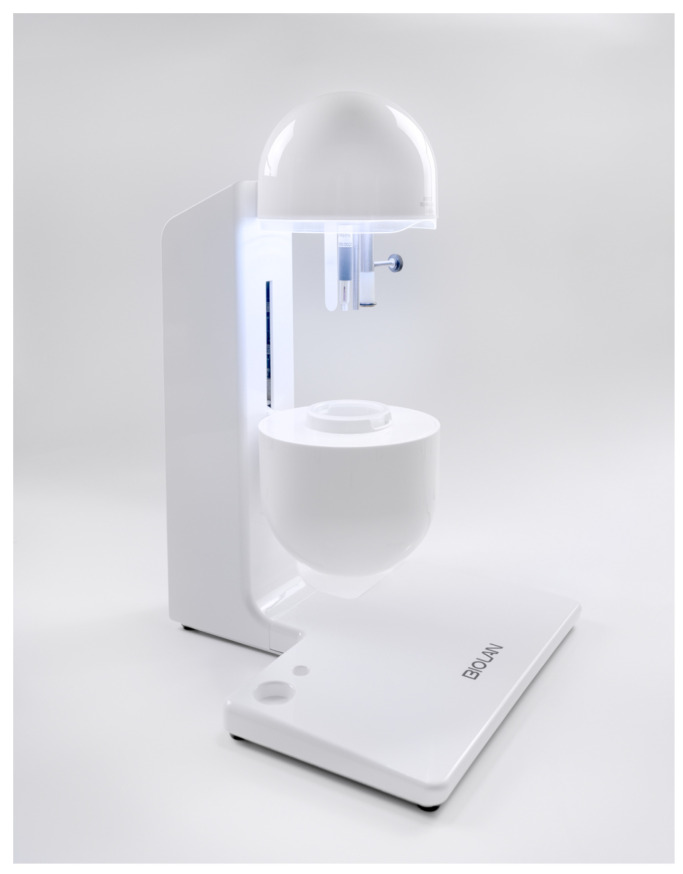
Benchtop enzymatic amperometrical biosensor from Biolan Microbiosensores.

**Figure 6:**
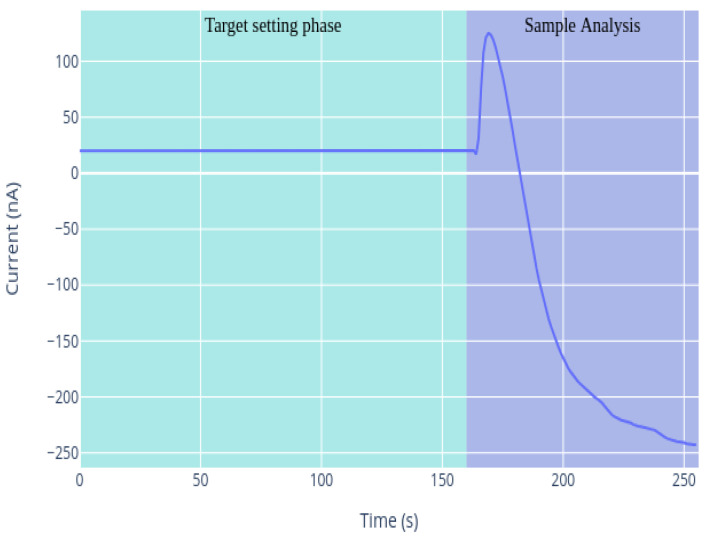
Different phases during sample analysis.

The measurements were taken with a benchtop enzymatic amperometrical biosensor from Biolan Microbiosensores (Figure 5). This technology is highly specific and selective and allows quantifying different food safety parameters in a short time (3–4 min). The biosensors currently marketed by Biolan are applied to food safety testing in different sectors, which are mainly for the fish, seafood and dairy sectors. The device combines the high specificity and selectivity of specific enzymes for each analyte to be measured with an amperometric transduction of the biological signal. Once the substrate is added to the cuvette, a change in the electric current is produced. Different analytes and samples can be tested with those devices depending on the application, for example, sulfite in crustaceans, histamine in raw fish and lactose or glucose in dairy products. The same electrochemical principle methodology is applied to measure the different analytes; only the enzyme is specific and different for each application. In all the procedures, two phases can be identified, the target phase and sample analysis (Figure 6). The data used in this work were provided by Biolan; all data were obtained from their laboratory where routine food safety testing is carried out. The different tests were carried out in the laboratory under controlled environmental conditions, where the temperature and the air of the room is controlled, and the same reagents were used to prepare the measuring solution needed to measure the concentration of the analyte of interest. Within the analyte of interest, different types of samples were tested to determine the reliability of the model.

For this study, 1700 amperometric measurements were collected using the named biosensor [19].

As mentioned above, amperograms are the amperometric transduction of the biological signal, the direct response of the enzymatic reaction. The total analysis of a sample (amperogram) consists of two phases, as shown in Figure 6.

The first phase is the adjustment to the target potential. an optimal potential is applied to the electrochemical cell; this electrode potential will be kept during the sample analysis. In this phase, the device records the electrical signal that circulates in the electrochemical cell (establishing the target) when there is no sample inside the cuvette. The optimal potential is the potential to which the electrochemical cell has to be adjusted, in such a way that the substance to be analyzed can donate or transfer electrons to the working electrode. This transfer is detected and translated into current. The optimal potential depends on the electroactive species analyzed, as the transfer of electrons has to be ensured. The device is programmed with this optimal potential, being an internal characteristic of the Biolan biosensor. During the first phase, the device records the electrical signal that circulates in the electrochemical cell (establishing the target) when there is no sample in the cuvette. In the target setting phase, the enzyme reaches stability, i.e., it is assured that the optimal conditions to proceed with the analysis of the sample are met, so that the enzyme works at full capacity. Once the first phase is finished, the device issues a sound signal, indicating that the target has been established correctly, so one can proceed with the second phase, the sample analysis. At this moment, the sample can be injected into the measuring electrochemical cell; the enzyme recognizes the analyte present in the sample; and its corresponding current is obtained due to the RedOx reaction.

Consequently, the idea of shortening this part was rejected, since it does not guarantee that the enzyme is stable, and this can lead to an inaccurate final measurement. Therefore, for the purpose of this work, only the second part was considered.

### 2.2. Data Preprocessing

First, preprocessing of the data was carried out, in which the data with some type of error such as inconsistent data or loss of signal were removed.

#### 2.2.1. Filtering Data

Amperogram measurements are signals that are registered every second. Since the signal obtained presented noise and disturbances, it was filtered to remove the noise. For this, a low pass and high pass exponential moving average (EMA) filter was used to eliminate the peaks present in data [20,21,22]. The EMA filtering consists of the following expression:

(1) $$A_{n}=\alpha \times M+\left(1-\alpha \right)\times A_{n-1}$$

The EMA filter presents a contribution of “new” information through the measurement *M* and a smoothing effect based on the memory given by the previous filtered value $A_{n-1}$. The result is a smoothed signal that depends on the factor α. This parameter is the filter constant with a value ranging 0≤α≤1. The decreasing of the α factor increases the smoothing of the signal. However, lowering the value of α could yield to erase some frequency components that are of interest, classifying as noise something that is actually a real variation of the signal. There is a trade-off between minimizing the noise and preserving the nature of the signal. In Section 3.2, the steps followed to find the optimal value of α are presented.

#### 2.2.2. Finding the Inflection Point of the Signal

Once the sample is injected into the cuvette, the sensor experiences changes in the current, and it takes time to stabilize; there is a moment of uncertainty in which the current presents random peaks. These few seconds may be due to the fact that, when the sample is injected, it needs time to homogenize in the medium; therefore, there are disturbances in the amperogram measurements. The homogenization process of the sample is considered to be stochastic, and the time taken for the enzymatic reaction to start randomly varies among samples. Hence, there is a need to find the point from which it is ensured that the enzymatic reaction has started and the amperogram presents the shape of an exponential function. Since it is a variable that cannot be controlled at first, it should not be taken into account as a parameter in the mathematical model. As a consequence, the enzymatic reaction was considered to start once the inflection point is reached, i.e., when the decay of the slope has started and the velocity of the reaction is the highest. Once the reaction begins, the current starts to drop exponentially, resembling an exponential function.

**Figure 7:**
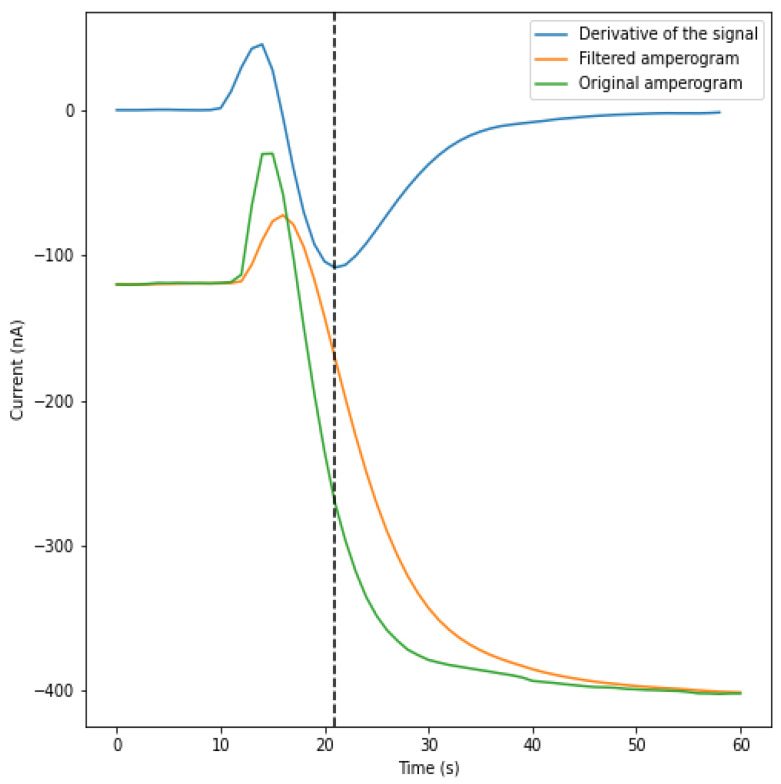
Finding the inflection point of the signal. The original signal, filtered signal and the derivative of the first order are represented. The inflection point obtained by the derivative is represented by the vertical dotted line.

One of the characteristics of exponential functions is that the minimum value of the function’s derivative is the point where the function starts to decay. Due to that, the point that is taken as the start point corresponds to the point at which the rate of the decrease of the reaction is maximum (Figure 7). This point in the derivative of the signal corresponds to the inflection point in the signal, which, in this case, corresponds to the minimum value of the derivative.

Due to the stochastic behavior of the signal once the sample is added in the cuvette, in some cases, the derivative of the signal has more than one inflection point. To avoid the initial peaks and obtain the optimal starting point while working in real time, if an increase in the signal is detected, a new inflection point is searched.

### 2.3. One Phase Exponential Model

The model was elaborated to illustrate the RedOx reaction that happens in the electrochemical cell when the sample is added.

**Figure 8:**
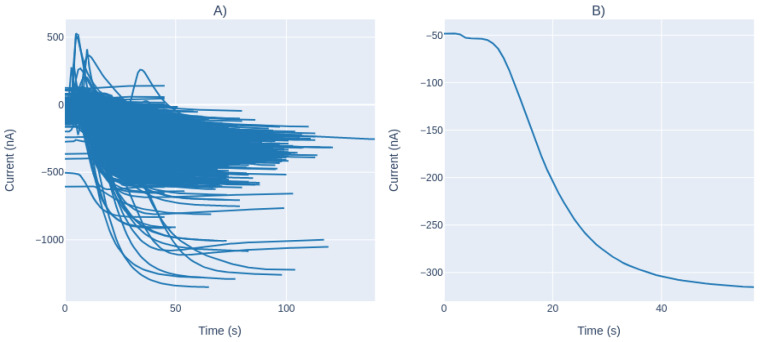
(**A**) Electric current during the analyte measurement on all the sample, with normalized data; and (**B**) electric current during a single measurement.

Figure 8A presents all the curves analyzed in the study; note that, these curves are normalized around the origin. In Figure 8B, a specific amperogram is presented, the measurement of the current resulting from the RedOx reaction. Changes in current are generated by the RedOx reaction produced in the cell. The resulting span is proportional to the bulk concentration of the analyte; thus, the reaction rate decreases due to the substrate consumption.

Figure 8B shows that, as the reaction progresses, the activity of the enzyme decreases as a result of the saturation stage that the substrate reaches. There is no free substrate in the medium, so the slope of the amperogram curve will no longer decrease and stabilize at the saturation point.

By observing all the measures, we concluded that the enzymatic reaction resembles an exponential decay reaction that could be modeled as a single exponential function by Equation (2):

(2) $$Current\left(t\right)=A\times e^{-B\times t}+C$$

*A*, *B* and *C* represent the characteristic coefficients of the sensor response: *A* represents the amount of analyte present in the medium, *B* is the decay constant that simulates the speed of the reaction and *C* is the term error for the least square fit accident.

The aim of this equation is to model the reaction of the enzyme with the analyte, once the starting point is found.

#### 2.3.1. Levenberg–Marquardt Algorithm

**Figure 9:**
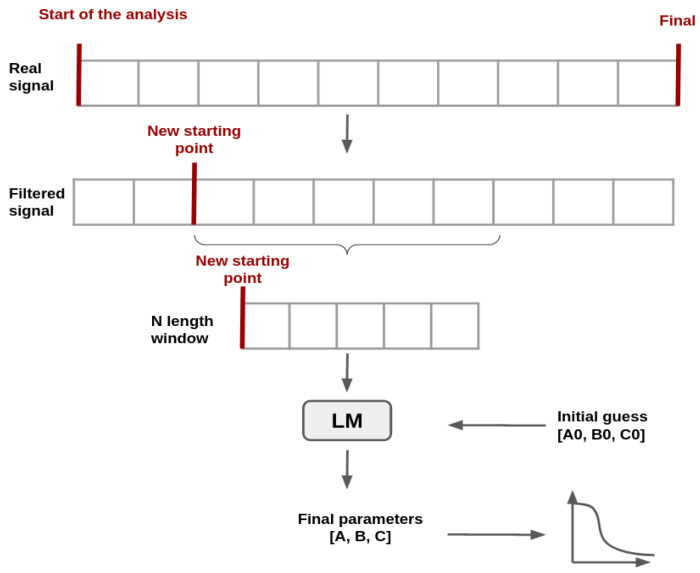
Diagram of the pipeline process.

The goal of the model is to determine values for parameters *A*, *B* and *C* presented in Equation (2) that make the curve best fit the sensor data. For that a nonlinear regression, algorithm was selected, named LM algorithm. The pipeline in Figure 9 summarizes the methodology used during the modeling process.

The LM method is used for fitting a parametric mathematical model to a set of data points by minimizing an objective expressed as the sum of the squares of the errors between the model function and a dataset. This method reduces the sum of the squares of the errors between the model function in Equation (2) and the data. LM method combines two minimization algorithms, the gradient descent method and the Gauss–Newton method.

It consists of solving Equation (3):

(3) $$\left(J^{t}\times J+\lambda \times I\right)\delta =J^{t}\times E$$

One of the characteristics of the LM algorithm is that the value λ is added to each member of the diagonal of the approximate Hessian Equation (4) before the system is resolved in order to see the direction of the gradient. Normally, the assigned value for the beginning of the algorithm is λ = 0.1, but it can take any value between 0 and 1.

(4) $$H\approx (J^{t}\times J)$$

Once the equation is solved, the estimated parameter values are updated by δ, and the obtained error is recalculated.

- If the new sum of squared errors has decreased, the value of λ is decreased.
- If the new sum of squared errors has increased, the new estimated parameters are discarded, and the method is repeated with a major value of λ.

The adjustment of λ is carried out with the adjustment factor, normally defined at 10. If λ needs to be increased it is multiplied by 10, whereas, if the value must decrease, it is divided by 10.

The steps are as follows:

1. Compute the Jacobian
  (5) $$J=\frac{\partial f_{i}\left(\beta \right)}{\partial \beta_{j}}\mathrm{where}\beta =\left(A,B,C\right)\to J=\left[\frac{\partial f}{\partial A}\frac{\partial f}{\partial B}\frac{\partial f}{\partial C}\right]$$
2. Compute the error gradient
  (6) $$g=J^{t}\times E$$
3. Approximate the Hessian Equation (4) and solve the LM Equation (3) in order to obtain the weight δ.
4. Update β {A,B,C} parameters using δ calculated in Step 3.
5. Recalculate the sum of squared errors using the updated weights:
  (a) If it has not decreased, discard new weights and increase λ and solve the LM Equation (3) again.
  (b) If it has decreased, decrease λ by 10.
6. If the error does not decrease anymore, end the iterations.

#### 2.3.2. Initial Guess

LM algorithm is an interactive algorithm; thus, the initial values of the model are essential. The goodness of the fit depends on the initial values. These initial values should be relatively close to the unknown parameters in order to avoid possible convergence problems. For that, a simple approach was done: the nonlinear model was transformed to a linear model, and the estimators for each parameter were solved in the transformed model [23].

### 2.4. Optimal Buffer Length Study

When dealing with temporal data, it is important to have enough information concerning previous values of the signal in order to make an appropriate decision about the shape of future data [24]. This size could be taken as all the data which have been recorded since the start of an operation or the last *n* arrived points [25].

It is worth noting that this *n* value is very important for the data analysis to be sound. When tackling temporal series, this size (*n* points) is called window size and refers to the size of a sliding window which is permanently monitored in order to detect the expected value, usually needed to make a certain decision [26].

**Figure 10:**
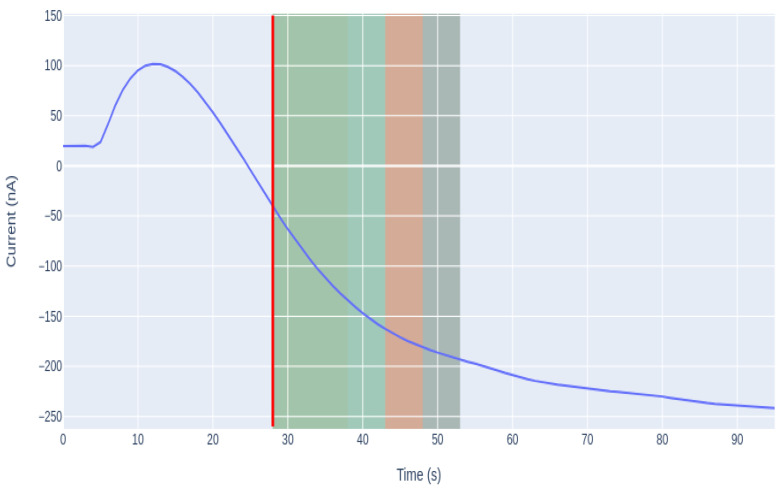
Example of the window size selected for the curve fitting. The window is chosen from the “new” starting point obtained with the minimum of the derivatives.

In this regard, the amount of information necessary in order to have a good approximation of the real curve was analyzed. For that, different lengths of buffers (widow sizes) were selected, beginning from the “new start point” obtained with the minimum of the derivatives (see Figure 10).

In Section 3.4, the process carried out to select the buffer size is detailed.

### 2.5. Model Evaluation

The variation of the percentage relative error (PE) [27] between estimated data and real data was studied according to Equation (7). It was used to report the difference between the experimental value and the estimated one, i.e. to quantify how close the approximation and the real value are.

(7) $$PE=\frac{I_{real}-I_{estimated}}{I_{real}}\times 100$$

## 3. Results and Discussion

In the previous section, the aspects to be taken into consideration are presented. In this section, the process carried out at each point and s the obtained results are shown.

### 3.1. Amperometrical Measurements Analysis

The mean duration of the analyses was studied, both the mean of the total duration of the analysis (taking into account both phases, namely target setting phase and sample analysis) and the duration of the sample analysis phase itself. The duration of all the periods is automatically determined by the control software of the biosensor. On average, the second phase, which corresponds to sample analysis, represents 49% of the total analysis.

**Figure 11:**
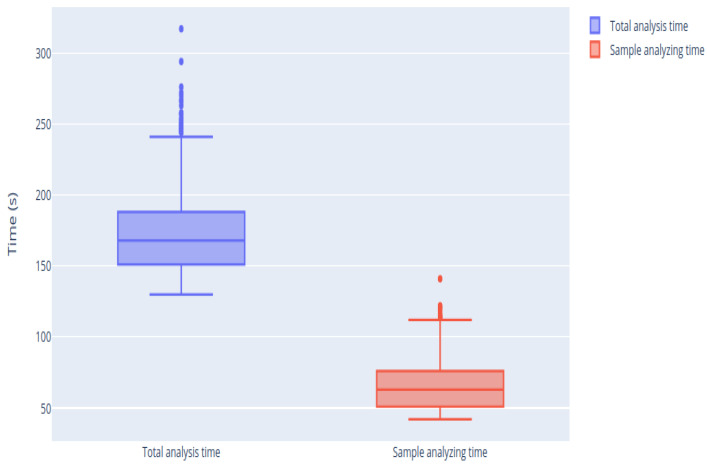
Box-plots of the duration of the different phases present in a sample analysis.

As shown in Figure 11, there is a dispersion throughout the whole analysis time of the measurement, since not all measurements take the same time to reach stabilization. This is because sometimes the signal is noisier or the reaction takes longer due to the biotest or the amount of analyte concentration.

### 3.2. Optimal Parameters for Filtering

Finding an optimal α for filtration is important: α impacts positively eliminating more noise and negatively delaying the signal. It is necessary to achieve a compromise between smoothing the signal and delaying it.

To obtain the optimal α, a sweep of different combinations was carried out: how this value affects the search for the inflection point of the signal was analyzed. Since, if the signal is not sufficiently smoothed, and the signal has too many spikes at the start due to the sample injection process, the derivative of the signal could have more than one peak, and it would be difficult to search its inflection point by the derivative in real time.

Table 1 summarizes the impact of delaying the signal with respect to the original. It was evaluated how it affects to the algorithm, and it was seen that a greater delay increases the mean error and decreases the amount of saved time, while there is no direct relationship with the number of curves where the algorithm was not applied. Those three characteristics were evaluated to obtain the optimal α. Table 1 summarizes the total of samples in which the algorithm was not applied (No algorithm), the percentage of samples in which the error was greater than 20% (% Misestimation), the mean error and the percentages of samples that had an error lower than the 10% with respect to the real value and an error lower than 20%, which are referred as Mean error (%), PE < 10% (%) and PE < 20% (%), respectively. It also summarizes the time saved in seconds by the algorithm with respect to the total analysis.

There are many reasons the algorithm is not applied in some samples: On the one hand, the alpha value is not adequate and it distorts the sample too much, so the algorithm is not capable of adjusting the curve. On the other hand, the reaction was fast and the saturation level was reached before the buffer was filled.

To evaluate the impact of α, a sweep of 20 parameter values was made, obtained by Equation (8):

(8) $$\alpha =\frac{2}{p+1}$$

and varying *p* in range [2, 20]. As shown in Table 1, the evolution of the variables to optimize (time and error) is proportional; therefore, a balance must be reached in a way that favors minimizing the error, even though time saving is penalized. To decide which alpha value is optimal, several considerations were taken into account. The minimum mean error value was sought; however, not only the minimum mean error but also how many samples fit with an error less than 20% was considered. Furthermore, it was intended for the misestimation to be as low as possible. Finally, it was considered that, in business terms, the number of “no algorithms” also needed to be low, as the more samples the algorithm is applied to, the more the costs are saved.

**Figure 12:**
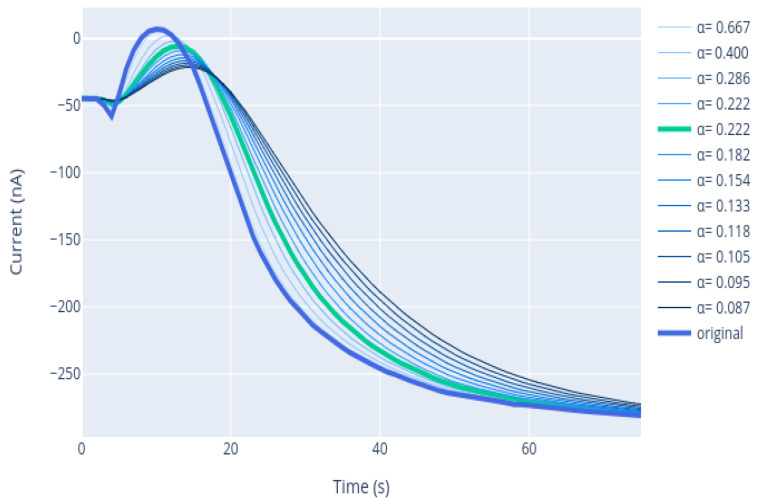
Finding the optimal parameter for filtering considering the influence of the filtering parameter on the original signal.

For the purpose of this work, it was considered that a parameter value of α = 0.22 is sufficient to smooth the signal and be able to find the optimal start point, highlighted in bold in Table 1. As shown in Figure 12, with this variable, we were able to smooth sufficiently the initial peaks to establish the new starting point. It was also considered that an adequate level of compromise was achieved between the variables to optimize, namely the time saved and the errors made estimating the signal’s last intensity.

### 3.3. Detection of the Starting Point of the Analysis

The injection of the sample into the measuring electrochemical cell results in an alteration in the signal. In some cases, an upper perturbation in the beginning of the amperogram can be observed, due to the sample or the way it was injected.

**Figure 13:**
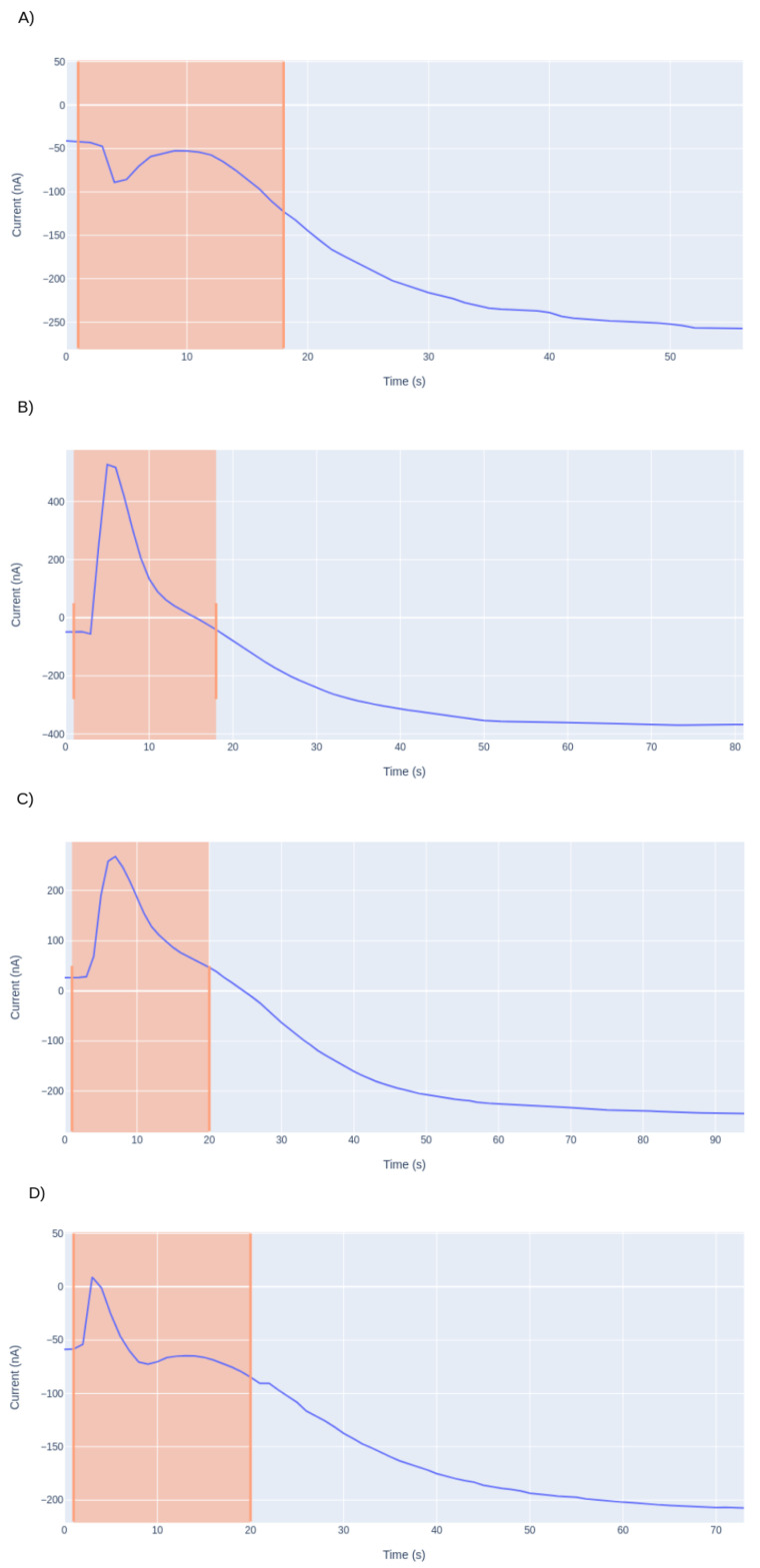
Different amperograms (**A**–**D**) with different types of behavior observed once the sample is injected.

**Figure 14:**
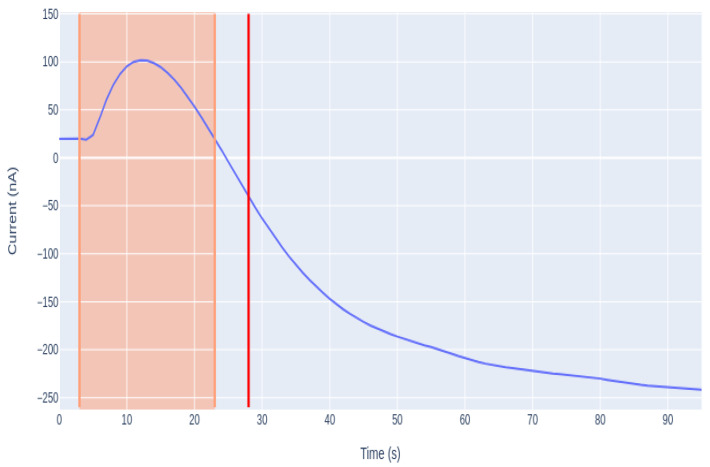
Graphical representation of the signal with the calculation of the point at which the derivative is minimum.

As shown in Figure 13, the transient period of time, in which the signal homogenizes in the medium and begins to fall, is different for each case. The presented amperograms are similar to an exponential signal, where the main goal is to quantify the span without waiting a long time for it to homogenize. To eliminate the random part of the signal, which is highlighted in pink, a derivative was performed. The derivative of the exponential implies the increment of it, so the minimum of the derivative is the starting point, which also corresponds to the beginning of the signal reduction. This derivative calculates the moment at which the signal begins to fall, thus is the point chosen as the new starting point of the analysis. In Figure 14, the new starting point is represented as the vertical red line.

### 3.4. Optimal Buffer Length for Curve Fitting

As mentioned in the previous section, an appropriate buffer (time window) size is crucial to get precise results. Various tests were carried out to obtain the optimal *n* (i.e., the number of data points) needed to get the proper parameters for the estimation of the last intensity of the amperogram [28]. Particularly, the amount of time the analysis can be shortened to obtain the minimum possible error when measuring the final intensity was considered.

**Figure 15:**
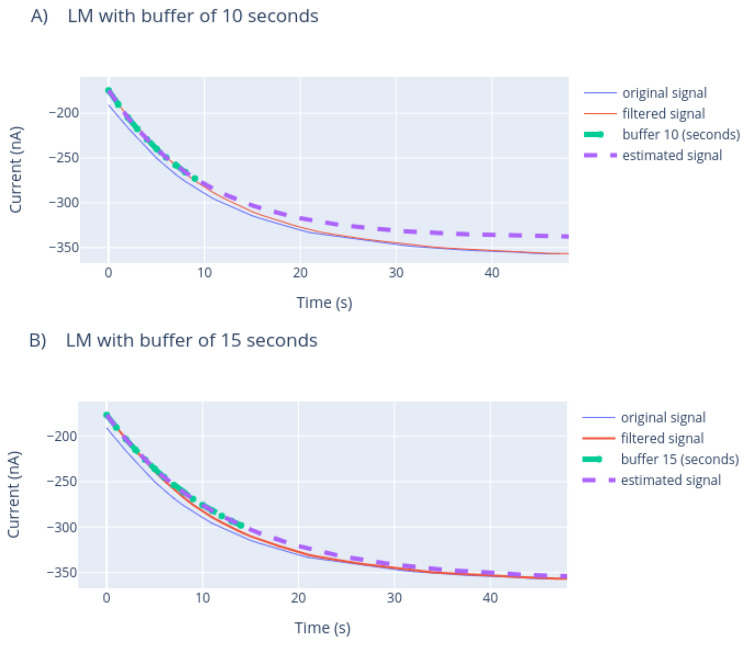

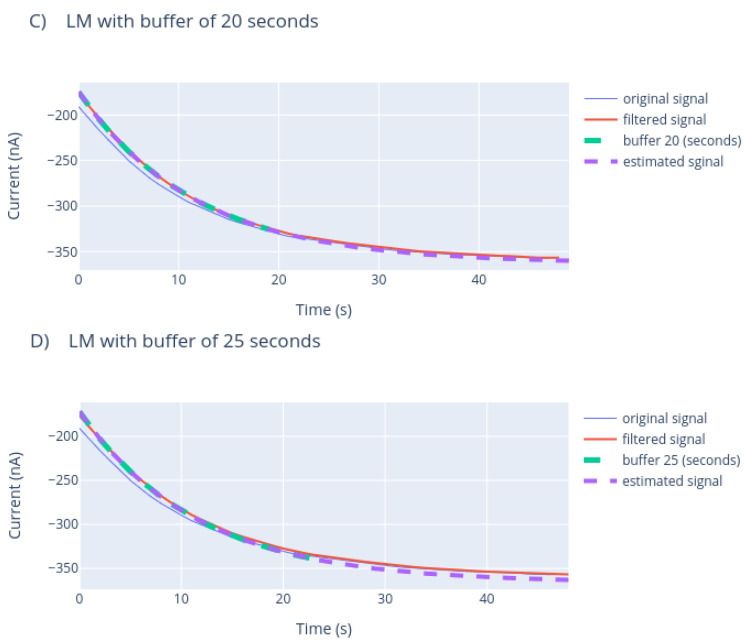
LM approach curve estimation with different buffer lengths. The length of the buffer corresponds to seconds.

Once the minimum number of data needed for the model to fit the data correctly was obtained, with the parameters estimated from the model, the sensor measurement data were simulated, as presented in Figure 15.

Figure 15 presents the estimation made with each of the window sizes, so a comparison can be made between the estimated value and the actual value measured by the biosensor.

### 3.5. Model Evaluation/Results

The goodness of fit of the model was analyzed with different window sizes. For that, the relative error between estimated data and real data was obtained by Equation (7), i.e., the error made between the last value of the analysis and the last model estimated value.

Table 2 summarizes the result obtained with different buffer lengths, the error made between the estimated and the actual value, and the mean value of saved time. The best results concerning the prediction error were given using a buffer of 20 s; however, the number of measurements where the algorithm is not applied and the saved time are also relevant when choosing the optimal buffer size. The number of curves where the algorithm was applied increases by 35% using a buffer size of 15 s and the saved time increases by 20%, while the mean error suffers an increase of only 12.3%. In this case, it is considered that the increment in the error term is low enough to be compensated by the improvement of the rest of the relevant parameters, so a buffer of 15 s was selected, highlighted in bold in Table 2.

**Figure 16:**
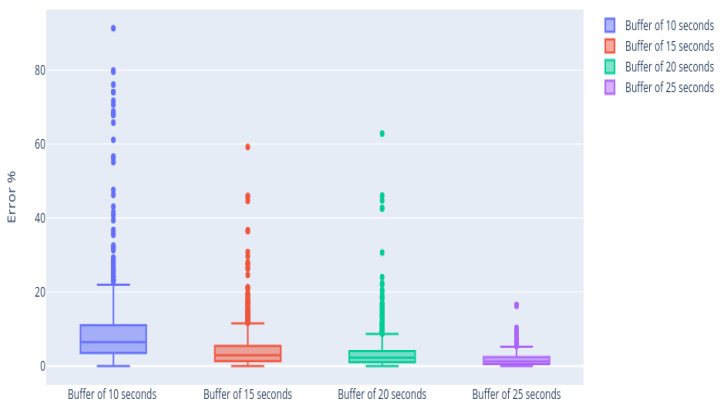
The box-plots of the error between the last real intensity of the amperogram and the estimated intensity.

Figure 16 shows that the distribution in each of the cases is uneven. As the length of the selected window increases, the error made in the estimation decreases: there is more information, so the curve fit is better. Increasing the buffer also means that the algorithm is not applied in all cases, since the analysis terminates before the buffer is full.

There are two reasons not to apply the algorithm: (1) when calculating the derivative and with the restrictions added to eliminate the random part of the beginning, the new starting point may be obtained almost at the end of the analysis; and (2) there are other cases where the reaction has been fast, and the stability has been reached, so the analysis has ended before filling all the buffer. Those two cases are labeled as “No algorithm” in Table 2.

**Figure 17:**
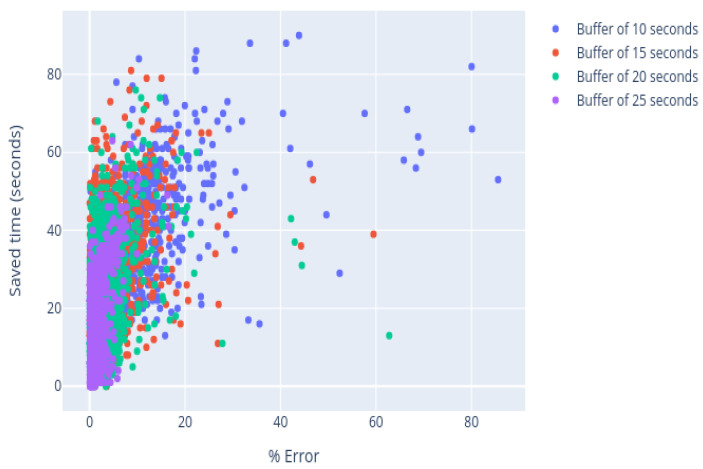
Saved time vs. error.

There is a compromise between the window size and the saved time, as can be seen in Figure 17. If the length of the buffer increases, the error is smaller, but some measures may also escape. Therefore, it is considered that a window of 15 is enough to fit the curve. Using these data, the algorithm can obtain the optimal parameters, and an average saving of 25 s can be obtained, i.e. 40% of the sample analysis duration.

**Figure 18:**
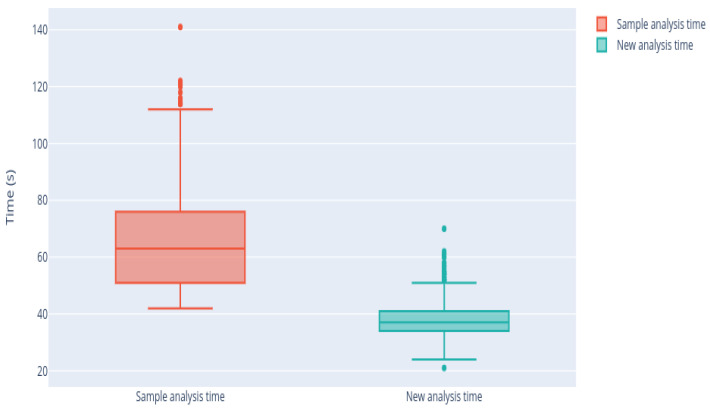
The box-plots of the analysis time and the time after applyinh the algorithm.

Figure 18 shows that this reduction is notable in all analyses. The average time of analysis with the traditional methodology is about 1 min, whereas, using the new proposed methodology, this time is reduced, obtaining an average analysis time below 40 s. The variability of the analysis time also decreased, that is, the analysis time is now more stable. The time variance was reduced from 267.32 to 36.98 s, showing that there is now greater stability in the duration of the measurements.

With all this, it is shown that the proposed methodology not only models the enzymatic reaction making it more dynamic, but also diminishes the variability of the measurement time obtained with the traditional methodology. If we evaluate the impact of the new methodology over the total analysis, taking into account both phases, it is observed that on average it is possible to reduce the time by 15%. If the times of all the measurements are added, it took a total of 63 h to perform all tests, whereas, if the algorithm had been used, it would have taken 53 h, a total saving of 10 h.

## 4. Conclusions

In this paper, a new approach is studied to evaluate the possibility of mathematically modeling the response of a biosensor to reduce measurement time. This study showed that the proposed exponential decay function can model accurately the enzymatic reaction that occurs within the electrochemical cell, thus it has the potential to reduce analysis time by estimating the last current value of the amperogram. In this work, the LM algorithm is used to estimate the parameters that describe the process and to model the reaction accurately. As a result, in 92% of the analyzed samples, the final value is estimated with an error lower than 10%. Furthermore, the proposed model can achieve a time reduction of 40% of the sample analysis duration, which results in the overall measurement time being reduced by 15%. This would lead to a more dynamic process and a reduction in costs for food industry.

The application of our proposed method should enable a faster obtaining of the biosensor measurement, keeping the error rate low enough to meet the food industry’s accuracy standards. To do so, it would be interesting to implement the methodology in a system working in real-time to test its performance in a real environment.

As future work, different issues are being tackled in order to improve the presented model: On the one hand, computational aspects could be improved aiming to obtain a faster classification of the sample and decide which window length would be enough to approximate the curve accurately. On the other hand, it is worth noticing that obtained curves could be different depending on the initial conditions of the test, and a previous categorization of different curves could be a previous step to select the appropriate method to deal with each known curve type.

## Figures and Tables

**Table 1:** Summary of parameter.

Name	α Filtration Parameter									
*p*	2	4	6	**8**	10	12	14	16	18	20
Parameter	0.6667	0.4	0.285	**0.22**	0.182	0.154	0.133	0.118	0.105	0.095
Total	1391									
No algorithm	36	25	31	**34**	36	38	39	39	47	53
Misestimation (%)	9.70	3.22	3.37	**2.58**	2.75	2.43	1.69	1.64	1.36	1.29
Mean error (%)	4.48	4.027	3.89	**3.74**	3.83	3.93	3.86	3.93	4.55	4.58
PE < 10% (%)	83.01	90.48	91.30	**92.13**	92.39	92.33	92.91	93.5	92.5	91.34
PE < 20% (%)	90.03	96.76	96.54	**97.34**	97.19	97.41	98.28	98.19	98.36	98.43
Saved time (s)	27.92	26.91	26.00	**25.09**	24.47	23.86	23.25	22.08	22.68	21.56

**Table 2:** Model results with different window size.

Window size	10	**15**	20	25
Total	1391			
No algorithm	64	**72**	111	649
Total algorithm	1327	**1319**	1280	742
Mean error (%)	7.716	**3.64**	3.24	1.77
Std error (%)	8.31	**4.73**	4	1.86
10% < PE < 20% (%)	20.34	**6.12**	3.35	0.4
PE < 10% (%)	74.83	**92.65**	95.85	99.59
Mean saved time (s)	32.85	**27.82**	23.1	16.109
Std saved time (s)	14.07	**13.75**	13.56	11.98

## Data Availability

Data available on request due to restrictions eg privacy or ethical.

## Abbreviations

The following abbreviations are used in this manuscript:

RedOx	Reduction-Oxidation
LM	Levenberg–Marquardt
EMA	Exponential Moving Average
$H_{2}O_{2}$	Hydrogen peroxide
$O_{2}$	Oxygen
$H_{2}O$	Water
HRP	Peroxidase
M	Chemical mediator
Ox	Oxidation
Red	Reduction

## References

[1] Vasilescu A., Fanjul-Bolado P., Titoiu A.M., Porumb R., Epure P. (2019). Progress in Electrochemical (Bio) Sensors for Monitoring Wine Production. Chemosensors.

[2] Amine A., Mohammadi H. (2018). Amperometry. Ref. Modul. Chem. Mol. Sci. Chem. Eng..

[3] Dodevska T., Lazarova Y., Shterev I. (2019). Amperometric Biosensors for Glucose and Lactate with Applications in Food Analysis: A Brief Review. Acta Chim. Slov..

[4] Martynko E., Kirsanov D. (2020). Application of Chemometrics in Biosensing: A Brief Review. Biosensors.

[5] Pisoschi A.M., Pop A., Gajaila I., Iordache F., Dobre R., Cazimir I., Serban A.I. (2020). Analytical methods applied to the assay of sulfur-containing preserving agents. Microchem. J..

[6] Xu K., Chen Q., Zhao Y., Ge C., Lin S., Liao J. (2020). Cost-effective, wireless, and portable smartphone-based electrochemical system for on-site monitoring and spatial mapping of the nitrite contamination in water. Sens. Actuators B Chem..

[7] Artigues M., Abellà J., Colominas S. (2017). Analytical parameters of an amperometric glucose biosensor for fast analysis in food samples. Sensors.

[8] Surya T., Sivaraman B., Alamelu V., Priyatharshini A., Arisekar U., Sundhar S. (2019). Rapid methods for histamine detection in fishery products. Int. J. Curr. Microbiol. App. Sci..

[9] Gao T., Tian Y., Zhu Z., Sun D.W. (2020). Modelling, responses and applications of time-temperature indicators (TTIs) in monitoring fresh food quality. Trends Food Sci. Technol..

[10] Ismail I., Oluleye G., Oluwafemi I. (2017). Mathematical modelling of an enzyme-based biosensor. Int. J. Biosens. Bioelectron..

[11] Parthasarathy P., Vivekanandan S. (2018). A numerical modelling of an amperometric-enzymatic based uric acid biosensor for GOUT arthritis diseases. Inform. Med. Unlocked.

[12] Domanskyi S., Privman V. (2017). Modeling and modifying response of biochemical processes for biocomputing and biosensing signal processing. Advances in Unconventional Computing.

[13] Da Silva D.C., Lopes S.M., de Aquino N.S.M., de Oliveira Elias S., Duda H.A., Tondo E.C. (2020). Mathematical modelling and validation of Salmonella enterica growth in sushi exposed to different time-temperature scenarios found in real sushi establishments. Food Res. Int..

[14] Qureshi S., Yusuf A., Ali Shaikh A., Inc M., Baleanu D. (2020). Mathematical modeling for adsorption process of dye removal nonlinear equation using power law and exponentially decaying kernels. Chaos Interdiscip. J. Nonlinear Sci..

[15] Chi F., Jiang B., Zhao Z., Chen Y., Wei X., Duan C., Yin M., Xu W. (2019). Multimodal temperature sensing using Zn_2_GeO_4_: Mn2+ phosphor as highly sensitive luminescent thermometer. Sens. Actuators B Chem..

[16] Caicedo N., Leturcq R., Raskin J.P., Flandre D., Lenoble D. (2019). Detection mechanism in highly sensitive ZnO nanowires network gas sensors. Sens. Actuators B Chem..

[17] Jouha W., El Oualkadi A., Dherbécourt P., Joubert E., Masmoudi M. (2018). Silicon carbide power MOSFET model: An accurate parameter extraction method based on the levenberg–marquardt algorithm. IEEE Trans. Power Electron..

[18] Mallick S.P., Dash D., Mallik S., Roshan R., Mahata S., Das P., Mahato S. (2017). An empirical approach towards photovoltaic parameter extraction and optimization. Sol. Energy.

[19] Biolan BIOLAN MICROBIOSENSORS, 2020. http://www.biolanmb.com/.

[20] Milici S., Lázaro A., Villarino R., Girbau D., Magnarosa M. (2018). Wireless wearable magnetometer-based sensor for sleep quality monitoring. IEEE Sens. J..

[21] Cui X., Bray S., Reiss A.L. (2010). Functional near infrared spectroscopy (NIRS) signal improvement based on negative correlation between oxygenated and deoxygenated hemoglobin dynamics. Neuroimage.

[22] Chugh S., Akula A. Effect of Different Signal Processing Techniques on a Calibration Free Pulse Oximeter. Proceedings of the 2018 3rd International Conference for Convergence in Technology (I2CT).

[23] Zhang G., Allaire D., Cagan J. An Initial Guess Free Method for Least Squares Parameter Estimation in Nonlinear Models. Proceedings of the ASME 2020 International Design Engineering Technical Conferences and Computers and Information in Engineering Conference.

[24] Cryer J.D. (1986). Time Series Analysis.

[25] Chou J.S., Ngo N.T. (2016). Time series analytics using sliding window metaheuristic optimization-based machine learning system for identifying building energy consumption patterns. Appl. Energy.

[26] Pinzón J.D., Colomé D.G. (2019). Real-time multi-state classification of short-term voltage stability based on multivariate time series machine learning. Int. J. Electr. Power Energy Syst..

[27] Tohidi-Hosseini S.M., Hajirezaie S., Hashemi-Doulatabadi M., Hemmati-Sarapardeh A., Mohammadi A.H. (2016). Toward prediction of petroleum reservoir fluids properties: A rigorous model for estimation of solution gas-oil ratio. J. Nat. Gas Sci. Eng..

[28] Shih S.Y., Sun F.K., Lee H.y. (2019). Temporal pattern attention for multivariate time series forecasting. Mach. Learn..

